# Saying no to drugs

**DOI:** 10.1007/s40037-015-0167-y

**Published:** 2015-03-27

**Authors:** Arul Thangavel, Bradley Sharpe

**Affiliations:** 1 Division of Internal Medicine, Department of Medicine, University of California, San Francisco, CA 94143-0320 USA; 2 Department of Medicine, University of California, San Francisco, USA

**Keywords:** Methamphetamine use, Electronic medical record, Patient narrative, Provider narrative

## Abstract

Illicit drug use, and the associated health problems, is a frequent issue encountered by medical providers. Because of its salience as a piece of health care information, drug use history is often included in patient charts and presentations by house staff and medical students. However, if used and recorded inappropriately, drug use history can have significant long-lasting effects on the perception of patients in a system where they have little agency.

It was my first day on my Obstetrics rotation, and I was flustered, running late and feeling damp from the blistering Fresno heat. I stepped through the hospital doors and wandered down a hallway replete with moans, yells and whimpers. I found the physician rounding room and glimpsed a white board divided into a grid, one line for each patient, rife with acronyms too arcane to decipher.

The chief resident saw me agape and assigned me my first patient, Ms. Lisa Valle^1^. I didn’t know what this assignment meant, but I understood enough to listen closely to the resident’s presentation. She quickly summarized the patient: ‘A 19-year-old G1P0 at 22 weeks. Methamphetamine user with PPROM on hospital day 12.’ The only word I understood was ‘methamphetamine,’ and I was petrified. I envisioned a large billboard back home in San Francisco that featured an emaciated woman, eyes glistening with psychosis, with only a lone tooth, depicting, hyperbolically, the disastrous effects of methamphetamine on the body.

The next morning I steeled myself for my first meeting with such a methamphetamine user. After reviewing Ms. Valle’s chart, I knocked, entered her room, and was taken aback. Here was a startlingly healthy woman. She had all of her teeth. She wasn’t emaciated. Disarmed, I stumbled through my questions—questions I’d prepared for a more hostile recipient. Lisa answered my questions calmly and asked important and pertinent questions about her baby’s health. As I went over her baby’s prognosis and the likely length of her hospital stay (not cheerful answers on either account) her demeanour paradoxically brightened a bit. She promised to think of more questions for me.

I learned more about her over the following week, including her ‘substance abuse history.’ She’d tried meth once years ago, a poor decision borne from late-night alcohol-fuelled peer pressure. When I first started my presentations in front of the entire team, I emulated my resident, pronouncing my patient’s drug history in my opening line. But eventually her methamphetamine use sank down through my presentation, until finally I didn’t mention it at all. It was a remote history written on the board, and even this seemed unjust.

Lisa’s drug history was a profound piece of health information for me. It was a perplexing thing; the word ‘methamphetamine’ scared me, but also set my mind whirring. If methamphetamines directly caused her condition, my cognitive response to her drug history could have been vital. But the relevance of remote, one-time drug use was more murky. The billboard’s emaciated, toothless spectre arose with little benefit to Lisa’s care.

After 2 weeks, Lisa’s condition had not changed. Each morning, at the end of her case discussion, a small pause ensued, as if to insulate such a hopeless patient from those more hopeful ones who came after. It was devastating to watch; my first ward as a someday doctor relegated to the corners of medicine, where treatment has yet to catch up with diagnosis.

Over the next few weeks, I visited Lisa frequently, watching her grow increasingly tired. One Wednesday, she was particularly alight, and when I asked after her good mood, she said she’d felt her baby moving. But when I asked the team about this hopeful turn, they were sceptical: the ultrasounds hadn’t shown any movement at all.

One morning, a classmate who had been on call the night before told me that Lisa’s temperature had risen and her blood pressure had dropped. Her baby had died and she had quickly turned septic. Her 25 weeks of pregnancy culminated in an emergent C-section. In the end, her baby had been doomed for weeks, a victim not of methamphetamine but a prematurely ruptured amniotic sac. As physicians pulled her baby from her, they saw the child had only a single, sad eye placed squarely in the centre of its head.

In clinic weeks later, I heard a voice call my name from across the hall. I looked up and saw Lisa standing on a scale with a medical assistant. I walked over, and she spoke animatedly about her time since leaving the hospital, with that same indefatigable cheerfulness I had encountered when we first met. She asked if I had heard what had happened to her child, and I said yes. I accompanied her into her appointment, and listened as the resident went through some compulsory questions and an examination. Her drug history came up once, the resident dutifully asking whether she was still using meth. A cloud passed over Lisa’s face. I realized sadly that she wasn’t privy to the prominent inclusion of her drug history in her daily presentations and discharge summary. Her sunny disposition faltered slightly on a confusion: is this what she was to people now?

In a medical system where patients have little agency, Lisa’s story highlights the power that health care providers wield. Lisa was no methamphetamine addict; her one-time drug use was tangential to her medical care. Yet, weeks later, ensconced in the medical record, this history haunted her care. In each medical appointment now, Lisa would explain to a new provider that methamphetamine addiction, for all its horrors, was not her burden to bear. Lisa’s story should remind us that we are patients’ stewards in an opaque system, and our behaviour, even at its most automatic and seemingly inconsequential, can profoundly affect their health care for years to come.

## Disclaimers

The views expressed in this essay are the author’s own and do not reflect the views of any educational body.

## Source(s) of support

None.

## Conflict of Interest declaration

The authors declare no conflict of interest.

## Footnotes

1 Name changed to protect identity of patient.

